# Seasonal difference in temporal transferability of an ecological model: near-term predictions of lemming outbreak abundances

**DOI:** 10.1038/s41598-018-33443-6

**Published:** 2018-10-15

**Authors:** Eivind Flittie Kleiven, John-André Henden, Rolf Anker Ims, Nigel Gilles Yoccoz

**Affiliations:** Department of Arctic and Marine Biology, UiT - The Arctic University of Norway, NO–9037 Tromsø, Norway

## Abstract

Ecological models have been criticized for a lack of validation of their temporal transferability. Here we answer this call by investigating the temporal transferability of a dynamic state-space model developed to estimate season-dependent biotic and climatic predictors of spatial variability in outbreak abundance of the Norwegian lemming. Modelled summer and winter dynamics parametrized by spatial trapping data from one cyclic outbreak were validated with data from a subsequent outbreak. There was a distinct difference in model transferability between seasons. Summer dynamics had good temporal transferability, displaying ecological models’ potential to be temporally transferable. However, the winter dynamics transferred poorly. This discrepancy is likely due to a temporal inconsistency in the ability of the climate predictor (i.e. elevation) to reflect the winter conditions affecting lemmings both directly and indirectly. We conclude that there is an urgent need for data and models that yield better predictions of winter processes, in particular in face of the expected rapid climate change in the Arctic.

## Introduction

Worries about the consequences of global environmental change have sparked calls for making ecology a more predictive science by increasing ecological models transferability in time and space^1–5^. While some studies still convey a pessimistic view on ecological models’ ability to predict complex ecosystem dynamics^6–8^, more optimistic views are emerging^9,10^. Hence, investigation of model transferability should be a priority in ecology^3,11–14^. Dietze *et al*.^13^ have just proposed that iterative cycles of near-term forecasting and validation (i.e. checks of model transferability) should be a systematic activity in ecological research - even in the initial stages of research and monitoring programs. A similar scheme (“adapting modeling”) has been proposed by Urban *et al*.^5^.

While most of the early ecological transferability literature has focused mainly on the predictive ability of abiotic environmental factors^15^, there is now an increasing understanding of the importance of including biotic mechanisms to improve forecasting ability^16–18^. Indeed, there is a growing body of both theoretical^19,20^ and empirical evidence^16,21^ for the importance of including biological interactions. The complexity of biotic interactions has been proposed to be one of the main challenges in forecasting ecosystem states^22,23^. Therefore, gaining knowledge about biotic interactions as basis for including them in predictive models has become an increasingly important issue. While a few case studies have investigated the transferability of models containing both biotic and abiotic predictor variables^24,25^, such validation studies are still few, in particular, for model projections that involve space for time substitution^26^.

Arctic ecosystems have been proposed to be a good starting point to investigate the temporal variability caused by biotic interactions due to their simplicity, i.e. low species diversity and relatively few biotic interactions^16^. A particularly promising case could be the trophic interactions in tundra ecosystems centered on the cyclic outbreak dynamics of lemming populations. These interactions have historically received much attention due to their general lesson for ecological theory and their key ecosystem functions^27–29^. An important attribute concerning the ecological functioning of the lemming cycle is the outbreak amplitude; i.e. the abundance of lemmings during peak phase of the outbreak cycle^30,31^. Lemming cycles exhibit considerable spatial and temporal variability in outbreak amplitude presumably due to the combined action of both abiotic (e.g. climatic) and biotic (intra - and interspecific density dependence) factors^32^. Two previous studies have shown that statistical models that both include abiotic and biotic factors predict quite well variation in outbreak amplitude of the Norwegian lemming (*Lemmus lemmus*) - either across space within a single outbreak^33^ or across several outbreaks for a single locality^34^. Here we investigate to what extent a model based on abiotic (elevation as a proxy for local climate) and biotic predictors (intra – and interspecific density dependence) of lemming outbreak abundances in space (i.e. Ims *et al*.^33^) has temporal transferability. Specifically, we do this by assessing the ability of a season-specific state-space model, parameterized by spatial data from one outbreak cycle to predict outbreak amplitudes in the subsequent cycle. By doing so we apply the framework of near-term forecasting of Dietze *et al*.^13^ to advance our knowledge about what causes variability in lemming outbreak abundance.

## Results

### Overall characteristics of the abundance dynamics

Comparing the two consecutive cyclic peaks, there were some differences in estimated abundances (Table 1). While estimated lemming abundances were similar in the two peak springs, the spatial variability in spring densities (cf. CV values in Table 1) was substantially larger in 2007 than in 2011. Moreover, the growth of the lemming population over the peak summer was higher in 2007 than in 2011 (Table 1), leading to 40% higher estimated autumn abundance in the first peak compared to the second peak. For the grey-sided vole that co-occur with the lemming in our study region, the estimated pre-peak autumn abundances were similar between the two peaks, while the estimated spring abundance was somewhat higher in the second compared to the first peak (Table 1).

**Table 1:** Estimates of grey-side vole and lemming abundance for the different seasons and years.

Year	Season	Grey-sided vole		Lemming	
		*λvole (sd)*	CV	*λlem (sd)*	CV
2006	Autumn	2.84 (2.49)	0.88	0.31 (0.65)	2.07
2007	Spring	0.70 (0.66)	0.94	0.47 (0.69)	1.48
2007	Autumn	3.41 (2.61)	0.77	2.16 (1.56)	0.72
2010	Autumn	2.51 (2.29)	0.92	0.35 (0.58)	1.67
2011	Spring	1.41 (1.17)	0.83	0.45 (0.14)	0.32
2011	Autumn	4.93 (4.22)	0.86	1.53 (0.99)	0.65

These abundances estimates are given as means and standard deviation (σ) of the posterior means (***λ***). Coefficient of variation (CV) quantifies the amount of spatial variability in abundances ($CV=\frac{\sigma }{\mu }$).

### Predictors of lemming peak abundances

The estimated coefficients of the fitted models for each of the two lemming peaks are given in Table 2. Lemming abundances were generally negatively affected by intra-specific density dependence. The elevation effect was consistently positive for the autumn abundances (i.e. increasing abundance with elevation). A positive effect of elevation was also found in the spring of the first peak, while there was no such over-winter effect during the second peak. Considering the interaction between the rodent species (inter-specific density dependence), there was a tendency for a positive effect of grey-sided vole autumn abundance on lemming spring abundance in the first peak, while we found no such effect in the second peak (credible intervals encompassed 0 with good margins; Table 2). The estimates for a grey-sided vole effects on autumn lemming abundance were smaller and less certain (credible intervals encompassed 0 with good margins; Table 2) in both peaks.

**Table 2:** Parameter estimates from the state-space model of season- and cyclic peak-specific lemming abundances, given as mean of the posterior distribution and 95% Bayesian credible intervals (CI).

Peak	Season	Inter-specific density dependence *β*_*dvole*_	CI	Elevation *β*_*alti*_	CI	Intra-specific density dependence (*β*_*dlem*_ − 1)	CI
2006/2007	Spring	0.267	[−0.270: 0.824]	0.845	[0.307: 1.439]	−0.999	[−1.143: −0.810]
	Autumn	0.100	[−0.227: 0.431]	0.528	[0.201: 0.857]	−0.909	[−1.162: −0.607]
2010/2011	Spring	−0.085	[−0.492: 0.254]	−0.134	[−0.477: 0.179]	−0.994	[−1.057: −0.812]
	Autumn	−0.090	[−0.407: 0.239]	0.390	[0.065: 0.795]	−0.350	[−1.624: 1.535]

One unit of the scaled elevation predictor is equivalent to 86 meter.

### Transferability

The temporally consistent parameter estimates of the autumn dynamics in both peaks contributed to a relatively good ability of the estimated autumn dynamics in 2007 to predict the site-specific autumn lemming abundances for 2011 (Fig. 2). The mean absolute error (MAE) was 0.55 individual per site for this model and there was no apparent bias (Fig. 2). In contrast, the spring part of the model parameterized by data from the first peak (2007) performed poorly in terms of its temporal transferability to the spring of the next peak (2011); as could be expected from the inconsistent estimates of the spring dynamics in the two peaks (Table 2). The MAE for the spring predictions was 0.70 individual per site and a strong bias was evident (Fig. 2). The MAE values have to be seen relative to the mean season specific abundance, which is more than 3 times higher in autumn than in spring. Therefore, the calculated MAE values show a clear difference in model transferability.

**Figure 1:**
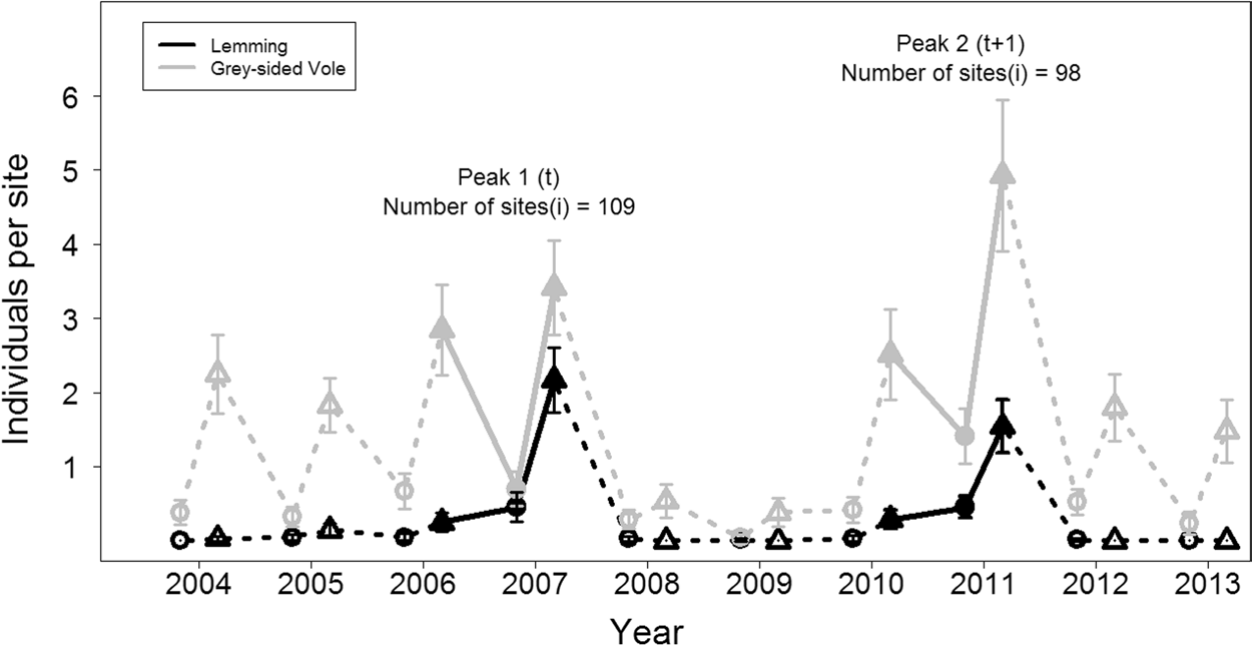
Population trajectories for Norwegian lemming and grey-sided vole displayed for the study area in north-easternmost Fennoscandia. The population trajectories are given as mean number of individuals trapped per site (with 2xSE bars) in spring (●) and fall (▲). The full line highlights the periods of the time series (i.e. the two cyclic outbreak phases) analyzed by the state-space model.

**Figure 2:**
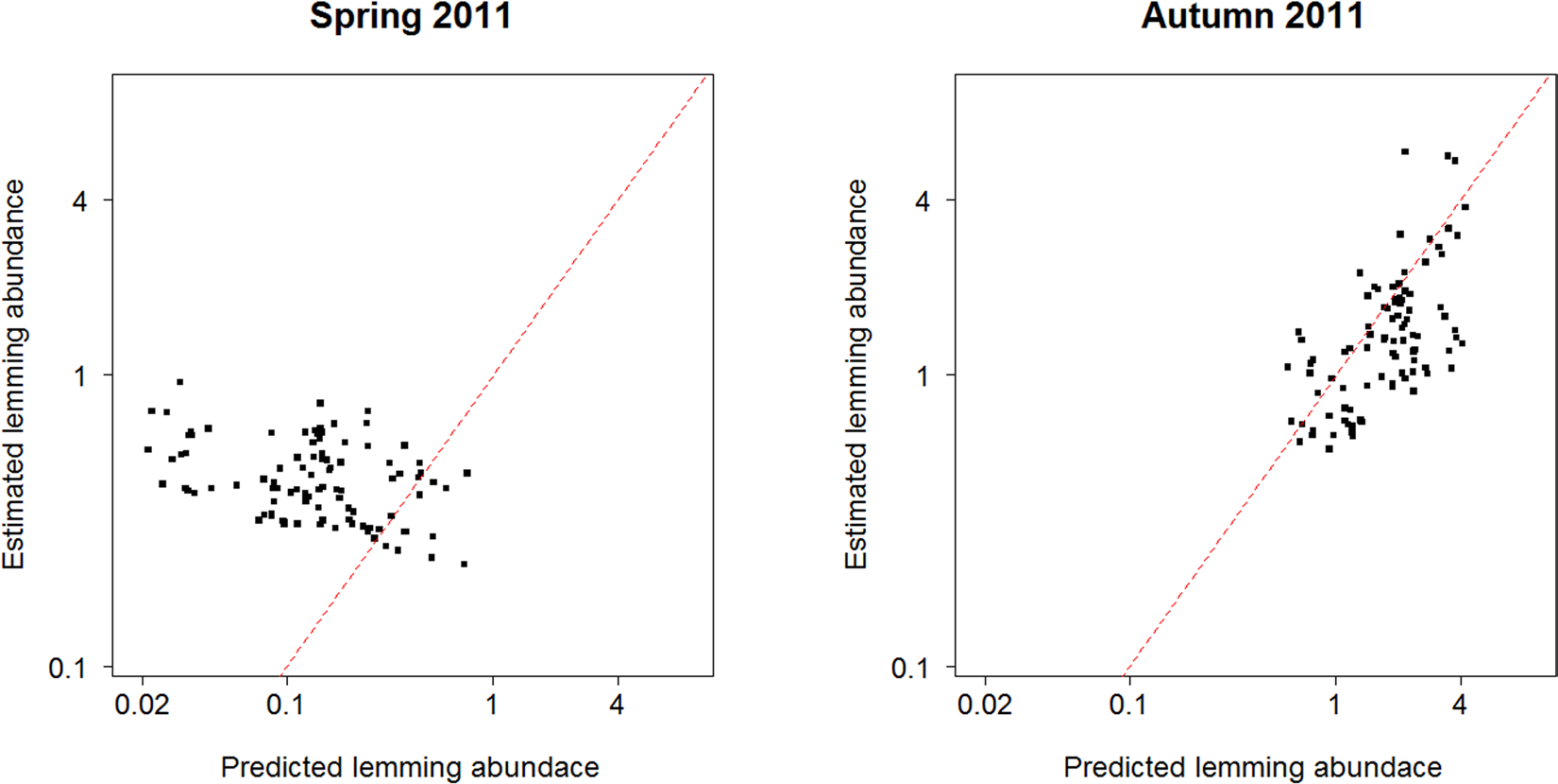
Graphical display of the season-specific (i.e. spring and autumn) temporal transferability of the lemming abundance on a logarithmic scale; i.e. the ability of the model parameterized with the data from the first peak (years 2006/07) to predict (x-axis) the estimated lemming site specific abundances (y-axis) in the second cyclic peak (years 2010/11). The red dotted line represents y = x.

## Discussion

We investigated the temporal transferability of a dynamical state-space model that was developed to identify season-specific biotic and abiotic predictors of cyclic lemming outbreaks. Based on spatial data from one lemming outbreak, Ims *et al*.^33^ found that a relatively simple model (i.e. with intra - and interspecific-density dependence and elevation as predictors) explained well the spatial variation in outbreak abundances. However, our results show that the temporal transferability of the model with respect to the subsequent cyclic lemming outbreak was only partial. That is, the model part projecting autumn abundances (i.e. reflecting population change over the summer) exhibited good transferability, whereas the model part predicting spring abundances (i.e. reflecting population change over the winter) performed poorly.

Previous studies have claimed that highly detailed knowledge about a modest number of interactions would be most beneficial regarding model transferability^35^. Indeed, our state-space model included few biotic interactions – i.e. only direct inter- and intra-specific density dependence – yet it appeared to be temporally transferable with respect to predicting population changes over lemming outbreak summers. This means that both the data (site-specific rodent abundance data and elevation) and the model appears adequate for near-term forecasting of lemming outbreak abundances in the autumn. However, the forecast of lemming spring abundances performed poorly, meaning that the data/model used for this purpose did not prove to be adequate/transferable.

This seasonal difference in the degree of model transferability is interesting. Arctic ecosystems are known to experience high temporal environmental variability both within (i.e. seasonality) and between years^36,37^. However, climatic variation between summers are known to be lower than between winters, in particular for the Atlantic sector of the Arctic^36^. Especially, large inter-annual differences in qualitative snow characteristics, towards which lemmings exhibit high sensitivity^34^, adds significantly to the winter variability. In particular, mild winters increase the hardness and humidity of the snow that impact lemming survival negatively^34^. Thus it appears that detailed temporal data and understanding of the impact of winter must be incorporated in models to provide temporally consistent predictions. Kausrud *et al*.^34^ did this with good result when they projected lemming dynamics for a single site. In the present study we attempted to make projection across a large number of sites within an area of approximately 10 000 km^2^ with elevation as a proxy of spatial climatic variation. Elevation has previously been used as a proxy for spatial variation in climate in many ecological studies^38^ including studies of outbreak amplitude of cyclic herbivore populations^39,40^. However, while elevation gradients may reflect spatial differences in snow conditions in some winters (i.e. winter 2006/2007), it may not in other winters (i.e. winter 2010/2011). Hansen *et al*.^41^ recently demonstrated that climatic extreme events during the winter in the high arctic could disconnect the association between snow quality and elevation. Generally, transferability of ecological models based on spatial data has been found to decrease whenever the magnitude and nature of the spatial and environmental variation differs between temporal domains^26,42^.

This study based on data from a relatively new program to monitor population dynamics of tundra rodents, should be seen as an initial loop of the iterative near-term forecasting cycle of Dietze *et al*.^13^. Thereby we have learned that the summer dynamics of outbreaking Norwegian lemming populations is near-term predictable based on the trapping data and elevation, whereas clearly more information is needed to be able to predict the pre-outbreak winter dynamics. We consider this lesson to be particular important in face of the ongoing rapid change in winter climate in the Arctic^43^.

## Methods

### Ethical statement

Rodent trapping was conducted as part of an ecological monitoring project that was initiated, financed and approved by The Norwegian Environmental Agency (NEA: ref no 06040003-4). NEA is the legal Norwegian authority that licenses sampling of all vertebrate wild life species for scientific purposes.

### System and sampling

The study was conducted within a tundra area of about 10 000 km^2^ at the north-easternmost tip of the Scandinavian Peninsula (70°N to 71°N). Rodent cycles with periodicity of 4-5 years prevail in the focal tundra ecosystem, with grey-sided voles (*Myodes rufocanus*) and the Norwegian lemming as the most abundant species^44^.

Since spring 2004, small rodent snap trapping has been performed on 98–109 sites according to the small quadrat method^45^, with one quadrat (i.e. 12 traps) per site (see Ims *et al*.^33^ for details). In order to include spatial variability in environmental conditions, the design contains trapping sites that span a range of 30 to 346 m.a.s.l. (mean of ~ 200 m.a.s.l.). The orographic effect of elevation amounts to a decrease of approximately 0.6 °C per 100 m^46^, making elevation a proxy for spatial variation in temperature. Trapping was conducted twice annually; 2 days in late June (spring) and two days in early September (autumn) before the onset of winter.

### Statistical modelling: analyses and validation

Following Ims *et al*.^33^, the trapping data were analyzed at the site level (*i*) including data for the two cyclic lemming peaks (*t* = 2 peaks) contained in the time series (see Fig. 1: i.e. i = 109 sites during the first peak in 2006–2007 and i = 98 sites during the second peak in 2010–2011). Typically, Norwegian lemmings are mostly absent in trapping data between peaks (e.g. Turchin *et al*.^47^, Fig. 1) thus we included only data from the pre-peak autumn (*k-2*) together with spring (*k-1*) and autumn (*k*) in the lemming peak years (*k* = 3 trapping seasons). The data for the two peaks were analyzed separately with the purpose of assessing the transferability of predictors of lemming outbreak abundance across different cycles. The predictors investigated were the same of those identified by Ims *et al*.^33^; elevation as a proxy for spatial climate variation and lemming and grey-sided vole abundance to model intra - and interspecific density dependence. The interspecific density dependence is most likely due to the influence of shared predators^33,48^. Ims *et al*.^33^ found that there was no residual spatial autocorrelation in the lemming abundance data so we did not include any extra spatial terms in the models. Moreover, previous time series analyses (e.g. Stenseth *et al*.^49^) have shown that there is no time-lags >2 years in small rodent population dynamics, meaning that consecutive cycles can be considered independent.

Small rodent trapping data includes stochastic sampling variability, therefore we analyzed the data using a state space model. We modelled the sampling variance in the number of trapped lemmings $({y}_{i,k,t})$ and grey-sided voles $({{\rm{x}}}_{{\rm{i}},{\rm{k}},{\rm{t}}})$ per site (*i*), season (*k*) and peak (*t*) as a Poisson process (λ)^33,49^. We used the mean absolute predictive error (MAE)^50^ to evaluate model fit (Appendix S1). We also plotted the estimated counts against observed counts to investigate whether there were some systematic differences between raw counts and estimated abundance.

With some small modifications from the model of Ims *et al*.^33^ (see Appendix S3), we then applied the following state-space model to estimate the season- (spring (k = 2) and fall (k = 3)) and peak outbreak-specific (year 2007 (t = 1) and 2011 (t = 2)) effects of elevation $({\beta }_{elev}$), inter-specific (${\beta }_{dvole}$) and intra-specific (${\beta }_{dlem}$) density dependence on lemming abundance:

$${y}_{i,k,t} \sim Poisson(\lambda {y}_{i,k,t})\,log(\lambda {y}_{i,k,t}) \sim Norm\,({\beta }_{{0}_{k,t}}+{\beta }_{dvol{e}_{k,t}}\ast log(\lambda {x}_{i,k-1,t})+{\beta }_{ele{v}_{i,t}}\ast ele{v}_{i,t}+{\beta }_{dle{m}_{k,t}}\ast log(\lambda {y}_{i,k-1,t}),{\sigma }_{t})$$

where *σ*_*t*_ is the standard deviation. For lemming abundance in the initial season (*k* = 1) and for grey sided vole abundance in all seasons (k = 1:3), the trapping data is also assumed to follow a Poisson process with mean λ_i,t_. However, since delayed effects cannot be included in the initial season, log (λ_i,t_) is modelled as Norm (*µ*_*i*,*t*_, *σ*_*t*_) where µ_i,t_ is a site-specific intercept. We checked that this difference between the autumn and spring models did not affect our conclusions regarding transferability, by fitting also a model with only the spring densities as a predictor of the fall densities (see Appendix S5).

Finally, to evaluate the temporal transferability, the model parameter estimates obtained based on data from the first peak (*t* = 1) was applied to the predictor data for the second peak (*t* = 2) to derive predicted lemming abundance ($p\lambda {y}_{i,k}$):

$$\mathrm{log}(p\lambda {y}_{i,k})={\beta }_{{0}_{k,t=1}}+{\beta }_{dvol{e}_{k,t=1}}\ast \,\mathrm{log}(\lambda {x}_{i,k-1,t=2})+{\beta }_{ele{v}_{k,t=1}}\ast ele{v}_{i,t=2}+{\beta }_{dle{m}_{k,t=1}}\ast \,\mathrm{log}(\lambda {y}_{i,k-1,t=2})$$

The predicted lemming abundance ($p\lambda {y}_{i,k}$) was validated against the estimated posterior means for lemming abundance for the second peak ($\lambda {y}_{i,k,t=2}$) by means of the mean absolute error^50^:

$$MAE=[{n}^{-1}{\sum }_{i=1}^{n}|({P}_{i}-\bar{P})-({O}_{i}-\bar{O})|],$$

with *P* being the predicted abundance ($p\lambda {y}_{i,k}$) and *O* the abundance estimated with the Poisson state-space model for the second peak ($\lambda {y}_{i,k,t=2}$). The mean ($\bar{P}\,and\,\bar{O}$) is subtracted to account for seasonal differences in abundance.

The state space models were specified in a Bayesian framework and priors were kept uninformative^51^. Posterior distributions were obtained using Markov Chain Monte Carlo (MCMC) techniques computed through Jags run from R (R Core Team 2015) using the jagsUI package. We used 4 chains, each of 50 000 iterations, with a burn-in of 15 000 (see Appendix S2 for details). To assess convergence of the chains, trace plots for all parameters where investigated graphically as well as from the Gelman-Rubin statistics (where $\hat{R}$ <1.1 indicates convergence)^52^.

## Electronic supplementary material

Supplementary Information

## Data Availability

All data analyzed in this paper is available on DRYAD, doi:10.5061.
