# Supplementary material for: Seasonal difference in temporal transferability of an ecological model: near-term predictions of lemming outbreak abundances
Source: Sci Rep. 2018 Oct 15;8:15252. doi: 10.1038/s41598-018-33443-6 (PMC6189055; doi:10.1038/s41598-018-33443-6)
Supplement: Supplementary file 1 — Supplementary Information [file 41598_2018_33443_MOESM1_ESM.docx]

**Seasonal difference in temporal transferability of an ecological model: near-term predictions of lemming outbreak abundances**

**Supplementary Information**

Eivind Flittie Kleiven (eivind.f.kleiven@uit.no), John-André Henden ([john-andre.henden@uit.no](mailto:john-andre.henden@uit.no)), Rolf Anker Ims ([rolf.ims@uit.no](mailto:rolf.ims@uit.no)) and Nigel Gilles Yoccoz ([nigel.yoccoz@uit.no](mailto:nigel.yoccoz@uit.no))

Department of Arctic and Marine Biology,

UiT - The Arctic University of Norway

NO – 9037 Tromsø

Norway

**Corresponding author:** Eivind Flittie Kleiven, Department of Arctic and Marine Biology, UiT - The Arctic University of Norway, NO, 9037, Tromsø, Norway. Email: [eivind.f.kleiven@uit.no](mailto:eivind.f.kleiven@uit.no).

# **Appendix S1:**

Mean absolute error has been found to be the most natural measure of average error magnitude and is described by the equation below ^1^.

$MAE=[n^{-1}\sum_{i=1}^{n} \left| {(P}_{i}-\bar{P} \right)- {(O}_{i}-\bar{O}) | ]$,

The mean ($\bar{P} and \bar{O}$) is subtracted to account for seasonal differences in abundance. As O we use the estimated abundance λ instead of raw counts because small rodent trapping data are known to be affected by stochastic sampling variability ^2^.

We calculate MAE to evaluate the internal predictability of the model (i.e. how well the model fit the data, see table S1-1).

Table S1-1: Estimated mean absolute errors (MAE) for evaluation of the internal predictability of the state-space model (i.e. how well the model fits the data).

| Species | Year | Season | MAE  (Individuals per site) |
| --- | --- | --- | --- |
| Lemming | 2006 | Autumn | 0.024 |
| Lemming | 2007 | Spring | 0.295 |
| Lemming | 2007 | Autumn | 0.758 |
| Lemming | 2010 | Autumn | 0.040 |
| Lemming | 2011 | Spring | 0.531 |
| Lemming | 2011 | Autumn | 0.730 |
| Grey-sided vole | 2006 | Autumn | 0.633 |
| Grey-sided vole | 2007 | Spring | 0.479 |
| Grey-sided vole | 2007 | Autumn | 0.615 |
| Grey-sided vole | 2010 | Autumn | 0.640 |
| Grey-sided vole | 2011 | Spring | 0.594 |
| Grey-sided vole | 2011 | Autumn | 0.766 |

Appendix S2:


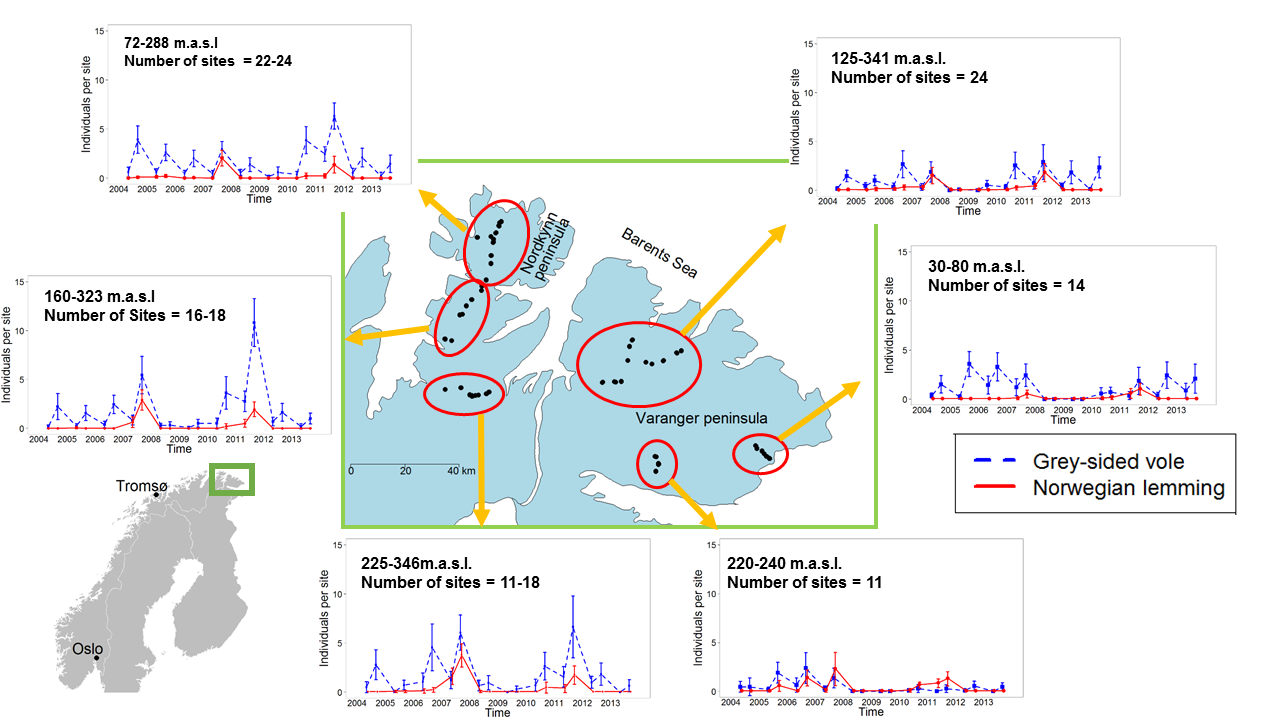


Figure S2-1: Population trajectories for Norwegian lemming and Grey-sided vole displayed for six sub-regions (circled in red) within the general study area in north-easternmost Fennoscandia. The population trajectories are based on the raw data and displayed as mean individuals trapped per site (with 2xSE bars) in spring and fall for each year. Black dots on the map denotes the trapping sites. The range of number of sites and their altitudes are shown for each sub-region.

The sampling design cover an area of more than 10 000km^2^. Figure S2-1 display the spatial distribution of the trapping sites. spatial auto correlation in the residuals was checked using moran I statistic by Ims et al. 2011^3^, but not found to be present. Hence the sites have been analyzed as independent spatial measurement.

The trapping sites within each of the 6 sub-regions is located along an elevation gradient to avoid regional confounding’s when using altitude as a proxy for spatial variability in climate.

# **Appendix S3:**

Jags model code.

model{

## 2006/07 ##

# observation process

for(i in 1:R){ #loop over all sites

for(k in 1:3){ # loop over all seasons

y_l07[i,k] ~ dpois(lambda_l07[i,k])

y_g07[i,k] ~ dpois(lambda_g07[i,k]) }}

# system process

for(i in 1:R){

for(k in 2:3){

mu_l07[i,k] <- beta0_07[k] + beta_vole1[k] * lambda_g07l[i,k-1] + beta_alti1[k] * alti_07[i]+ beta_dd1[k]*lambda_l07l[i,k-1] }

for(k in 1:3){

lambda_l07l[i,k] ~ dnorm(mu_l07[i,k], tau_l07[k])

lambda_l07[i,k] <- exp(lambda_l07l[i,k]) }}

# priors for mu2

for(i in 1:R){

for(k in 1:3){

lambda_g07[i,k] <- exp(lambda_g07l[i,k])

lambda_g07l[i,k] ~ dnorm(mu_g07[k],tau_g07) }}

# priors

for (i in 1:3) {

tau_l07[i] <- pow(sigma_l07[i],-2)

sigma_l07[i] ~ dunif(0,10) }

tau_g07 <- pow(sigma_g11,-2)

sigma_g07 ~ dunif(0,10)

for(i in 1:R){

mu_l07[i,1] ~ dnorm(0,0.01) }

for(k in 1:3){

mu_g07[k] ~ dnorm(0,0.01) }

for(k in 2:3){

beta0_07[k] ~ dnorm(0,0.01)

beta_vole1[k] ~ dnorm(0,0.01)

beta_alti1[k] ~ dnorm(0,0.01)

beta_dd1[k] ~ dnorm(0,0.01) }

## 2010/11 ##

# observation process

for(i in 1:R2){

for(k in 1:3){

y_l11[i,k] ~ dpois(lambda_l11[i,k])

y_g11[i,k] ~ dpois(lambda_g11[i,k]) }}

# system process

for(i in 1:R2){

for(k in 2:3){

mu_l11[i,k] <- beta0_11[k] + beta_vole2[k] * lambda_g11l[i,k-1] + beta_alti2[k] * alti11[i]+ beta_dd2[k]*lambda_l11l[i,k-1] }

for(k in 1:3){

lambda_l11l[i,k] ~ dnorm(mu_l11[i,k], tau_l11[k])

lambda_l11[i,k] <- exp(lambda_l11l[i,k]) }}

# priors for mu2

for(i in 1:R2){

for(k in 1:3){

lambda_g11[i,k] <- exp(lambda_g11l[i,k])

lambda_g11l[i,k] ~ dnorm(mu_g11[k],tau_g11) }}

# priors

for (i in 1:3) {

tau_l11[i] <- pow(sigma_l11[i],-2)

sigma_l11[i] ~ dunif(0,10) }

tau_g11 <- pow(sigma_g11,-2)

sigma_g11 ~ dunif(0,10)

for(i in 1:R2){

mu_l11[i,1]~dnorm(0,0.01) }

for(k in 1:3){

mu_g11[k] ~ dnorm(0,0.01) }

for(k in 2:3){

beta0_11[k] ~ dnorm(0,0.01)

beta_vole2[k] ~ dnorm(0,0.01)

beta_alti2[k] ~ dnorm(0,0.01)

beta_dd2[k] ~ dnorm(0,0.1) }

#### Predicting next peak ###

for(i in 1:R2){

for(k in 2:3){

lambda_pr[i,k] <- beta0_07[k] + beta_vole1[k] * lambda_g11l[i,k-1] + beta_alti1[k] * alti11[i]+ beta_dd1[k]*lambda_l11l[i,k-1] }}}

**Appendix S4:**

The State-space model presented in this paper is similar to the state-space model used in Ims et al. (2011) with a few differences.

However, there are a few minor differences. There are two vole species present in the study area, grey-sided vole and tundra vole (*Microtus oeconomus*), of which the first is usually the most abundant and widely distributed. Ims et al. (2011) used the sum of the two small rodent species as the predictor of inter-specific density-dependence effects of lemming abundance. While most of the trapped voles was grey-sided vole in the peak of 2007, tundra vole was trapped in substantially larger numbers in the peak of 2011 (Figure S4-1). Since the effect of interspecific density-dependence in Ims et al. (2011) mainly comes from grey-sided vole, we choose to include only grey-sided vole in the present model.


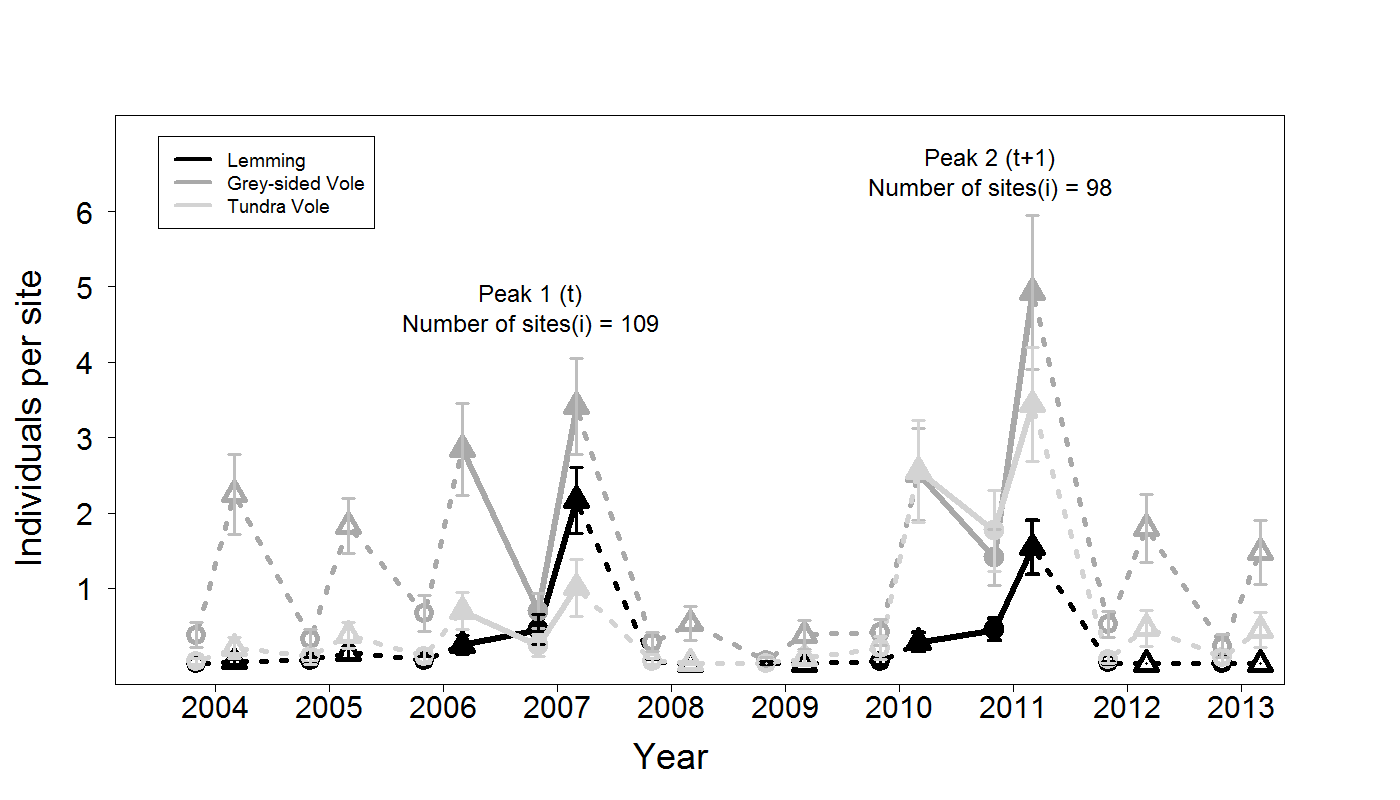


Figure S4-1: Population trajectories for Norwegian lemming, grey-sided vole and tundra vole displayed for the study area in north-easternmost Fennoscandia. The population trajectories are given as mean number of individuals trapped per site (with 2xSE bars) in spring(●) and fall(▲). The full line highlights the periods of the time series (i.e. the two cyclic outbreak phases) analyzed by the state-space model.

Ims et al. (2011) used vole abundance the previous spring to predict lemming abundance both the subsequent autumn and spring and by means of separate models for the two seasons. Here we used the same dynamical model for both seasons where the model was always parametrized with vole data from the previous season (i.e. the previous vole autumn data for predicting lemming spring abundance and previous vole spring data for predicting lemming autumn abundance).

Note that these deviations did not appear to affect the main conclusions in the study regarding the different degree of model transferability of winter and summer dynamics.

**Appendix S5:**

As one can see in appendix S3, pre-peak autumn (k=1) lemming abundance is estimated without predictor variables, while lemming abundances in peak spring (k=2) and peak autumn (k=3) has 3 predictor variables (elevation, inter-specific $\beta_{\mathrm{dvole}}$and intra-specific density dependence). To make sure that this difference does not affect the estimates of seasonal model transferability, we fitted a model for just the peak spring and peak autumn (k=2 and k=3), where lemming abundance in the peak spring was estimated without predictor variables (identical to season k=1 in the initial model), see jags code below. This did not affect the estimated MAE of the autumn model.

Jags code: note that k=1 is now the spring in the peak year and k=2 is the autumn in the peak year. i refer to number of sites (i=109 (R) in first peak and i=98 (R2) in second peak)

*## 2006/07 ##*

*#observation process*

*for(i in 1:R){ #loop over all sites*

*for(k in 1:2){ # loop over all seasons*

*y_l07[i,k] ~ dpois(lambda_l07[i,k])*

*y_g07[i,k] ~ dpois(lambda_g07[i,k])*

*}}*

*# system process*

*for(i in 1:R){*

*mu_l07[i,2] <- beta0_07 + beta_vole1 * lambda_g07l[i,1] + beta_alti1 * alti_07[i]+ beta_dd1*lambda_l07l[i,1]*

*for(k in 1:2){*

*lambda_l07l[i,k] ~ dnorm(mu_l07[i,k], tau_l07[k])*

*lambda_l07[i,k] <- exp(lambda_l07l[i,k])*

*}}*

*# priors for mu2*

*for(i in 1:R){*

*for(k in 1:2){*

*lambda_g07[i,k] <- exp(lambda_g07l[i,k])*

*lambda_g07l[i,k] ~ dnorm(mu_g07[k],tau_g07)*

*}}*

*# priors*

*for (i in 1:2) {*

*tau_l07[i] <- pow(sigma_l07[i],-2)*

*sigma_l07[i] ~ dunif(0,10)*

*}*

*tau_g07 <- pow(sigma_g11,-2)*

*sigma_g07 ~ dunif(0,10)*

*for(i in 1:R){*

*mu_l07[i,1] ~ dnorm(0,0.01)*

*}*

*for(k in 1:2){*

*mu_g07[k] ~ dnorm(0,0.01)}*

*beta0_07 ~ dnorm(0,0.01)*

*beta_vole1 ~ dnorm(0,0.01)*

*beta_alti1 ~ dnorm(0,0.01)*

*beta_dd1 ~ dnorm(0,0.01)*

*## 2010/11 ##*

*#observation process*

*for(i in 1:R2){*

*for(k in 1:2){*

*y_l11[i,k] ~ dpois(lambda_l11[i,k])*

*y_g11[i,k] ~ dpois(lambda_g11[i,k])*

*}}*

*# system process*

*for(i in 1:R2){*

*mu_l11[i,2] <- beta0_11 + beta_vole2 * lambda_g11l[i,1] + beta_alti2 * alti11[i]+ beta_dd2*lambda_l11l[i,1]*

*for(k in 1:2){*

*lambda_l11l[i,k] ~ dnorm(mu_l11[i,k], tau_l11[k])*

*lambda_l11[i,k] <- exp(lambda_l11l[i,k])*

*}}*

*# priors for mu2*

*for(i in 1:R2){*

*for(k in 1:2){*

*lambda_g11[i,k] <- exp(lambda_g11l[i,k])*

*lambda_g11l[i,k] ~ dnorm(mu_g11[k],tau_g11)*

*}}*

*# priors*

*for (i in 1:2) {*

*tau_l11[i] <- pow(sigma_l11[i],-2)*

*sigma_l11[i] ~ dunif(0,10)*

*}*

*tau_g11 <- pow(sigma_g11,-2)*

*sigma_g11 ~ dunif(0,10)*

*for(i in 1:R2){*

*mu_l11[i,1]~dnorm(0,0.01)*

*}*

*for(k in 1:2){*

*mu_g11[k] ~ dnorm(0,0.01)}*

*beta0_11 ~ dnorm(0,0.01)*

*beta_vole2 ~ dnorm(0,0.01)*

*beta_alti2 ~ dnorm(0,0.01)*

*beta_dd2 ~ dnorm(0,0.1)*

*#### Predicting next peak ###*

*for(i in 1:R2){*

*lambda_pr[i,2] <- beta0_07 + beta_vole1 * lambda_g11l[i,1] + beta_alti1 * alti11[i]+ beta_dd1*lambda_l11l[i,1]*

*}*

**References**

1 Willmott, C. J. & Matsuura, K. Advantages of the mean absolute error (MAE) over the root mean square error (RMSE) in assessing average model performance. *Climate Research* **30**, 79 (2005).

2 Stenseth, N. C. *et al.* Seasonality, density dependence, and population cycles in Hokkaido voles. *Proc. Natl. Acad. Sci. U. S. A.* **100**, 11478-11483 (2003).

3 Ims, R. A., Yoccoz, N. G. & Killengreen, S. T. Determinants of lemming outbreaks. *Proc. Natl. Acad. Sci. U. S. A.* **108**, 1970-1974 (2011).
